# Does GnRH treatment at TAI regardless of estrus occurrence increase pregnancy rate in crossbred *Bos taurus* suckled cows?

**DOI:** 10.1590/1984-3143-AR2023-0123

**Published:** 2024-03-15

**Authors:** Vanessa Silva Fernandes, Gabriella dos Santos Velho, Mateus Felipe Osório dos Santos, Kelly Alves Evangelista, Bernardo Garziera Gasperin, Luiz Francisco Machado Pfeifer, Rogério Ferreira, Paulo Bayard Dias Gonçalves, Gustavo Desire Antunes Gastal, André Gustavo Cabrera Dalto, Monique Tomazele Rovani

**Affiliations:** 1 Setor de Grandes Ruminantes, Universidade Federal do Rio Grande do Sul - UFRGS, Porto Alegre, RS, Brasil; 2 Programa de Pós-graduação em Veterinária, Universidade Federal de Pelotas - UFPEL, Pelotas, RS, Brasil; 3 Empresa Brasileira de Pesquisa Agropecuária - Embrapa, Porto Velho, RO, Brasil; 4 Departamento de Zootecnia, Universidade do Estado de Santa Catarina - UDESC, Chapecó, SC, Brasil; 5 Universidade Federal de Santa Maria - UFSM, Santa Maria, RS, Brasil; 6 Estación Experimental La Estanzuela, Instituto Nacional de Investigación Agropecuaria - INIA, Colonia, Uruguay

**Keywords:** GnRH, estrus, ovulation, conception rate, timed artificial insemination

## Abstract

The impact of GnRH treatment on the day of TAI in beef cows has received limited investigation, especially concerning its association with estrus expression. Consequently, two experiments were conducted to assess the potential of GnRH treatment on the day of TAI to enhance fertility according to the expression or not of estrus in beef cows. Experiment 1 aimed to determine ovulation rate and luteal function, while Experiment 2 aimed to determine the effect of the two GnRH treatment approaches on pregnancy rate. In Experiment 1, multiparous Brangus suckling cows (n = 17) were submitted to an 8-day TAI protocol. Estrus occurrence was evaluated based on chalk removal on D10 (TAI) and cows were assigned to receive GnRH (25µg lecirelin; im) according to the group: GnRH (n = 7), regardless of estrus expression; or selectGnRH (n = 10), only cows not detected in estrus. Ovulation rate occurring until 77h after IVD removal did not differ (p = 0.17) between GnRH (85.7%; 6/7) and selectGnRH (100%; 10/10). Also, corpus luteum size and serum progesterone concentration were not affected (p>0.05) by treatments. In Experiment 2, crossbred taurine suckled cows (n = 384) were submitted to the same protocol as described in Experiment 1 and were randomly allocated to GnRH or selectGnRH groups. There was no difference in P/AI between groups (selectGnRH = 55.6%; GnRH = 54.3%; p = 0.7) 30 days after TAI. As expected, there was a pronounced effect (p<0.0001) of estrus expression on P/AI (Estrus = 61.5%; No estrus = 33.0%), regardless of group. In summary, ovulation timing and rate and luteal function did not differ between groups. Also, GnRH administration only in cows that do not show estrus is recommended, considering hormone savings and similar conception rate.

## Introduction

Artificial insemination (AI) promotes the genetic evolution of a herd, being favored by the use of estrous cycle synchronization, mainly through the use of progesterone (P4) intravaginal device (IVD) associated with estradiol esters or GnRH (Bó et al., 2013). In Brazil, protocols based on P4 and estradiol are the most used in beef cattle (Bó et al., 2013), and the use of estradiol cypionate (EC) as an ovulation inducer brings the advantage of reducing one day of cattle handling, as it is injected on the day of IVD removal, compared to estradiol benzoate (EB), which is usually injected 24 h after IVD removal (Colazo et al., 2003). However, the use of this hormone is associated with the artificial expression of estrus (Pfeifer et al., 2020) and with the dispersion of ovulation (Souza et al., 2009).

The occurrence of estrus at the time of AI is one of the factors associated with the fertility of cows subjected to the TAI protocol (Richardson et al., 2016; Consentini et al., 2023). An alternative method to assess estrus expression is the use of chalk or paint markers at the tail base to identify females with greater or lesser estrus behavior at the time of AI (Nogueira et al., 2019). GnRH has been recommended to increase pregnancy per AI (P/AI) in cows that do not show estrus based on removal of the tail chalk at TAI (Cedeño et al., 2021). Furthermore, studies performed with *Bos indicus* cows showed that the use of GnRH at TAI increased the fertility in cows that did not express estrus (Madureira et al., 2020) and even increased the P/AI in cows that expressed estrus by the time of TAI (Prata et al., 2020). Alves et al. (2021) showed that P/AI can increase by more than 10 percentual points when GnRH is administered at TAI in *Bos indicus* cows. The authors observed a greater effect in cows that did not show estrus, primiparous, with a body condition score lower than 3 (1 to 5 scale), and cows treated with 0.5mg of EC. However, the benefit of using EC at IVD withdrawal followed by GnRH treatment in all cows at TAI has not yet been compared to the administration of GnRH only in animals not detected in estrus. Thus, the hypothesis of the present study was that GnRH treatment in all cows hastens ovulation and increases pregnancy rate compared to treatment only in cows that do not show estrus.

Two experiments were carried out to evaluate if GnRH treatment at TAI in all cows, regardless of estrus occurrence (GnRH group), would improve fertility compared to selective GnRH treatment (selectGnRH group), in which GnRH is administered only in cows that did not show estrus up to 48 h after IVD removal. The objective of Experiment 1 was to determine ovulation rate and luteal function, while the objective of Experiment 2 was to determine the effect of the two GnRH treatment approaches on pregnancy rate.

## Methods

The procedures described below were approved by the Ethics Committee on Animal Use - UFRGS (No. 41037). Experiment 1 was carried out at Agricultural Experimental Station - UFRGS, and Experiment 2 was conducted in 7 farms in Rio Grande do Sul and Santa Catarina states (Southern Brazil).

### Experiment 1

To evaluate ovulation rate and luteal function, multiparous suckling Brangus cows (n = 17), aged 3 to 5 years with > 40 days postpartum, were submitted to the following TAI protocol: D0, cows received 2 mg EB i.m. (RIC-BE, Agener União) and IVD (1 g of P4, Sincrogest, Ourofino); D8, the IVD was removed, and cows received 0.5 mg of EC i.m. (Cipiotec, Agener União), 0.52 mg of sodium cloprostenol i.m. (PGF; Estron, Agener União), 300 IU equine chorionic gonadotropin i.m. (eCG; Novormon, Zoetis), and tail chalk (Walmur marker stick) at the base of the tail to monitor estrus; D10 (48 h after P4 removal), cows received 25 µg of lecirelin i.m. (GnRH; Tec-Relin, Agener União) according to the group (GnRH or selectGnRH) and the evaluation of estrus expression was carried out by the same evaluator. Expression of estrus was considered when ≥ 75% of chalk was removed.

Cows were randomly allocated to two groups: selectGnRH: only cows not detected in estrus received GnRH (n = 10); or GnRH: all cows received GnRH, regardless of estrus (n = 7). The preovulatory follicle (POF) was monitored by transrectal ultrasonography (DP-10Vet, Mindray with a 5 MHz linear probe) 54 h, 72 h and 77 h after IVD removal. Ovulation was defined as the disappearance between assessments of a previously identified follicle greater than or equal to 8 mm in diameter.

Diameter (cm), circumference (cm), and area (cm^2^) of the corpus luteum (CL) were evaluated five days (D5) after GnRH treatment (48 h after IVD removal). In case of cavitary CL, two measurements were made (total and cavity) and the two values were subtracted to obtain a representative value of the CL parenchyma (Pugliesi et al., 2016). In addition, blood was collected from all cows by puncturing the caudal coccygeal vein using a 25 x 0.7 mm needle attached to a vacutainer tube with clot activator on D5 and D13 after GnRH treatment. Samples were centrifuged at 2500 rpm for 5 min. The extracted serum was placed in 1.5 mL microtubes and stored at -20 °C until P4 concentration analysis was carried out using the chemiluminescence method using the Elecsys Progesterone III Cobas Assay (Roche Diagnostics, Mannheim, Germany; REF 07092539) with sensitivity of 0.21 ng/mL and an intra-assay CV of 3.1%.

### Experiment 2

Crossbred taurine suckled cows (n = 384, > 40 days postpartum) were submitted to the same TAI protocol described in Experiment 1 to evaluate P/AI. Estrus detection was performed at TAI (48 h from the IVD removal). The animals were randomly allocated to: selectGnRH (only cows not identified in estrus received GnRH; n = 198) and GnRH (cows received GnRH, regardless of estrus; n = 186) groups. Pregnancy diagnosis was performed 30 days after AI using transrectal ultrasonography. Pregnancy losses were evaluated 90 days after TAI in a subgroup of animals (n = 173) from 5 different farms.

### Statistical analysis

Continuous data were analyzed for distribution and normalized using the Shapiro-Wilk test when necessary. CL measurement data and serum P4 concentration at different times were compared using mixed model. The pregnancy rate was compared using logistic regression and the contrast was used for comparisons of P/AI proportions according to the manifestation of estrus. Farm and bull had no significant effect and therefore were excluded from the model. Pregnancy losses between 30 and 90 days of pregnancy were compared between groups in a subgroup of animals using chi-square test. The analyzes were carried out with the JMP statistical software (JMP Statistical Discovery LLC.) and considered at a minimum significance level of 5% for the established comparisons.

## Results

### Experiment 1

**Figure 1:**
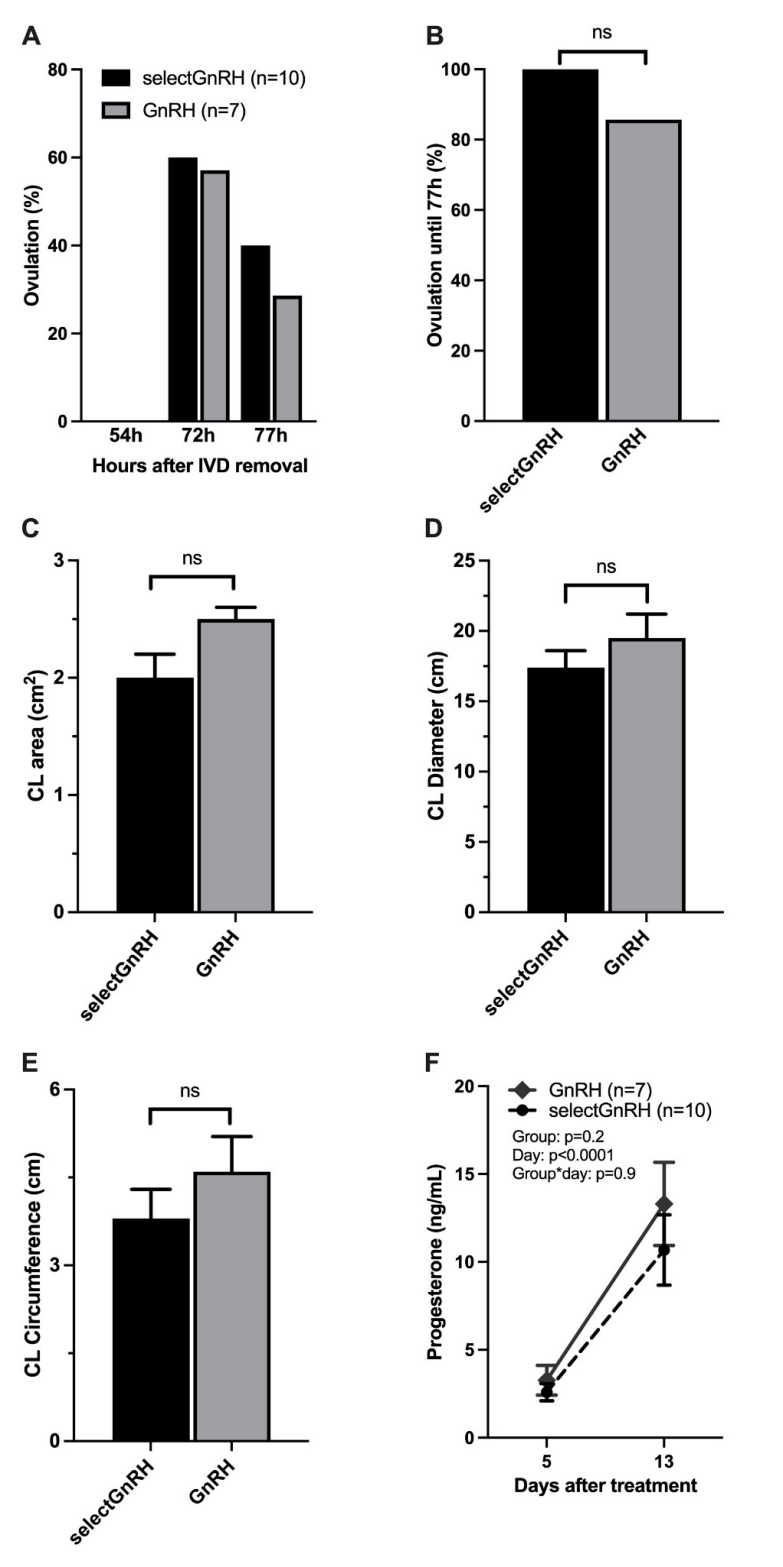
Ovarian assessments (A-E) and progesterone (P4) profile (F) in suckled Brangus cows in selectGnRH (GnRH-treated if not identified in estrus; n = 10) and GnRH (all GnRH-treated; n = 7) groups treated 48 hours after removal of the intravaginal progesterone device (IVD). Ovulation rate 54h, 72h and 77h after P4 removal (A) and cumulative ovulation rate up to 77 hours after P4 removal (B) in selectGnRH and GnRH groups. Corpus luteum (CL) area (C); diameter (D); and circumference (E) on day 5 after GnRH treatment. Progesterone concentration (ng/mL) on days 5 and 13 after GnRH treatment (F). ns = not significant.

The POF diameter did not differ between selectGnRH (10.1 ± 0.8 mm) and GnRH (10.5 ± 0.9 mm) groups 54 h after P4 removal (p > 0.7). No ovulations were observed at 54 h after P4 removal, regardless of group. However, 6 cows in the selectGnRH group (60%) and 4 cows in the GnRH group (57.1%) ovulated at 72 h; while at 77 h, 40% (4/10) of the cows in the selectGnRH ovulated and only 28.6% (2/7) of the cows in the GnRH group ovulated (Figure 1A). Therefore, the ovulation rates up to 77 h after P4 removal did not differ (P > 0.05) between groups, with 100% (10/10) for selectGnRH group and 85.7% (6/7) for GnRH group (Figure 1B). The CL area (p = 0.3; Figure 1C), CL diameter (p = 0.6; Figure 1D) and CL circumference (p = 0.4; Figure 1E) did not differ between groups. Serum progesterone concentration increased (p = 0.0001) from D5 to D13, but there was no difference between selectGnRH and GnRH groups (p = 0.2), and no group x day interaction (p = 0.9; Figure 1F).

### Experiment 2

**Figure 2:**
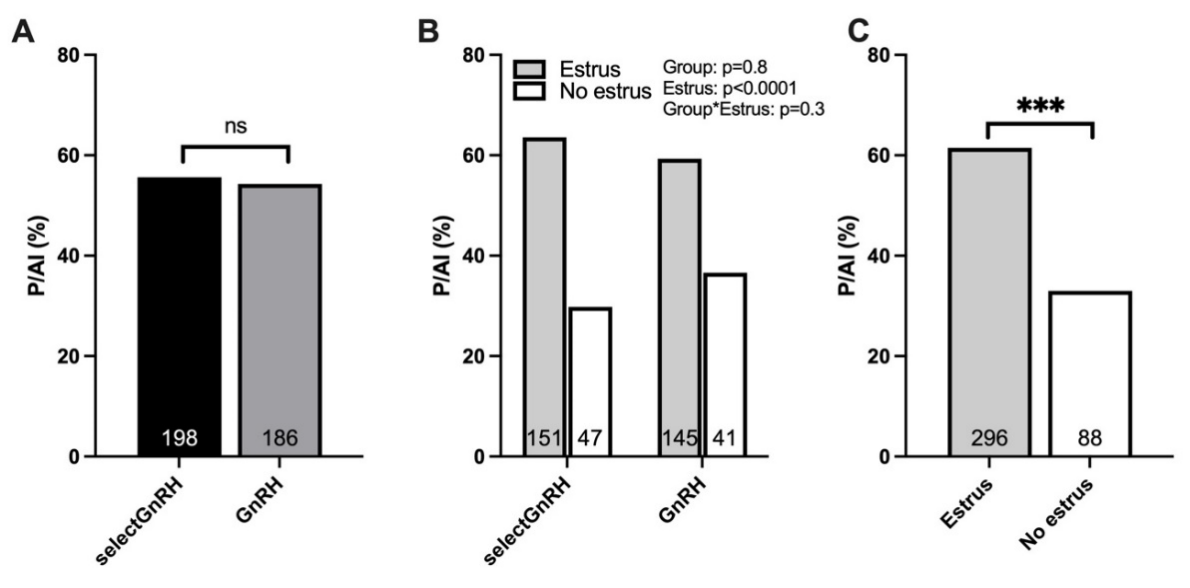
Pregnancy rate (P/AI) in crossbred taurine suckled cows after treatments with selectGnRH (GnRH-treated if not identified in estrus; n = 198) and GnRH (all GnRH-treated; n = 186) groups. P/AI in selectGnRH and GnRH groups (A); P/AI in selectGnRH and GnRH groups according to estrus expression at TAI (B); and P/AI according to the expression of estrus at TAI moment, regardless of the group (C). ns = not significant; ***represents p<0.001; numbers on the bars represent the number of animals.

The pregnancy rate (P/AI) did not differ (p = 0.7) between the GnRH (54.3%) and selectGnRH (55.6%) groups (Figure 2A). Considering that the groups were balanced according to estrus manifestation at 48 h, there was no significant effect of group on estrus occurrence (P=0.8; Figure 2B). The P/AI was higher (p < 0.0001) for cows that expressed (61.5%) compared to those that did not express estrus (33%), regardless of the group (Figure 2C). Pregnancy losses between 30 and 90 days of pregnancy were 4.7 and 4.9% for selectGnRH and GnRH groups, respectively (P = 0.96).

## Discussion

The present study compared two approaches regarding GnRH treatment at TAI in *Bos taurus* crossbred cows. Most of the cows ovulated between 72 h and 77 h after P4 removal, regardless of the group, and all cows in the selectGnRH group ovulated until 77 h. Moreover, no difference was observed in POF diameter, nor in CL area, diameter and circumference, and P4 concentration between GnRH and selectGnRH groups. Other studies that induced ovulation with EC alone observed ovulations from 48 h to 72 h in *Bos taurus* cows (Pancarci et al., 2002) and from 48 h to 96 h in *Bos indicus* cows (Sales et al., 2012). However, when GnRH was used as ovulation inducer, the cows ovulated from 24 h to 32 h after treatment (Pursley et al., 1995). Therefore, the results from our study indicate that, when EC is administered at IVD removal, GnRH treatment at TAI in all the cows does not advance nor improve ovulation synchronization, compared to the selective treatment (only in cows that did not express estrus).

In the present study, the results from ovulation and luteal function were corroborated by pregnancy rates, which did not differ between groups. Previous studies demonstrated that GnRH treatment in cows with small preovulatory follicles negatively affects pregnancy rate (Perry et al., 2005, 2007). Although follicular diameter at GnRH treatment was not evaluated, no negative effect was observed in the current study when all the cows received GnRH. This may be explained by the fact that the main difference between groups was the GnRH administration in cows that were in estrus in the GnRH group (but not in selectGnRH group), which likely are the cows with larger preovulatory follicles (Perry et al., 2007) and those that ovulate earlier (Cedeño et al., 2021).

Cows that showed estrus at the time of AI receiving or not GnRH, had higher P/AI than those that did not show estrus and received GnRH. Similar results were observed in Zebu cows, without the use of GnRH (Sá et al., 2010) and with or without GnRH in all cows (Prata et al., 2020). However, in the current study, all cows that did not show estrus at the time of AI received GnRH, regardless of the group. Thus, it is not possible to isolate the effect of GnRH treatment in cows that did not express estrus. Regarding the time of GnRH administration, we have demonstrated in a previous study that GnRH treatment in Nelore cows 34 h after IVD removal increased P/AI in cows that expressed or not estrus until the moment of GnRH treatment (Barbosa et al., 2022). However, in that study GnRH was administered 14 h before TAI. Prata et al. (2020) found similar results in protocols using P4 for 7 or 8 days, in which GnRH at the time of AI had a positive effect on the fertility of Nellore cows, including those that expressed estrus at the time of AI.

The use of GnRH in cows that do not show estrus until 48 h after IVD followed by TAI at 56 h increased P/AI (Cedeño et al., 2021), whereas no effect was observed when GnRH treatment and TAI were performed at 48 h in *Bos taurus* crossbred cows. Taken together, results from the current and previous studies suggest that future studies should focus on strategies to improve estrus manifestation and/or to improve pregnancy rates in cows that do not show estrus, as proposed by Cedeño et al. (2021).

## Conclusion

The use of GnRH treatment at TAI in all cows versus only in cows without estrus manifestation does not improve ovulation synchronization, luteal function, and pregnancy rate in *Bos taurus* crossbred cows. Therefore, the selective use of GnRH only in cows that do not show estrus is recommended, considering hormone savings and similar conception rate.
